# Taking cues from convalescence to improve vaccines against hepatitis C virus

**DOI:** 10.1172/JCI161819

**Published:** 2022-08-01

**Authors:** Bharath K. Sreekumar, Taha Y. Taha, Melanie Ott

**Affiliations:** Gladstone Institute of Virology, University of California San Francisco, Chan Zuckerberg Biohub, San Francisco, California, USA.

## Abstract

Hepatitis C virus (HCV) infection remains a worldwide public health issue despite direct-acting antivirals. A substantial proportion of infected individuals (15%–45%) spontaneously clear repeated HCV infections with genetically different viruses by generating broadly neutralizing antibodies (bNAbs). However, translating this response into an effective vaccine strategy has been unsuccessful. In this issue of the *JCI*, Frumento and colleagues report on their study of bNAb evolution longitudinally in convalescent individuals with repeated infections. Using pseudotyped viruses, well-characterized monoclonal antibodies, and complex modeling, the authors show that multiple exposures to antigenically related, antibody-sensitive viral envelope proteins induced potent bNAbs. This work provides valuable insight into the best strategies for developing HCV vaccines in the future that may successfully reproduce the immunity induced during natural exposures.

## Challenges for HCV vaccine development

Hepatitis C virus (HCV), a bloodborne, positive-strand RNA virus of the family *Flaviviridae,* infects the liver and can establish life-long infection and progressive liver inflammation. Despite the remarkable success of recent direct-acting antiviral (DAA) therapies, it remains a worldwide problem, with 58 million people chronically infected and about 1.5 million acquiring the infection each year (1). The majority of deaths are caused by liver cirrhosis and hepatocellular carcinoma, which occur at a rate of 15% to 30% within 20 years after infection. Several critical barriers hinder the control of this disease, including the following: (a) the majority of infected individuals do not know that they are infected due to lack of initial symptoms or access to diagnostics (of all estimated infections, only 21% were diagnosed in 2019) (1); (b) DAAs are expensive and inaccessible for high-risk individuals, such as intravenous drug users and prisoners (only 62% of diagnosed patients were treated in 2019 worldwide); and (c) DAA therapy does not prevent reinfection. Therefore, the development of an effective HCV vaccine that prevents and/or resolves chronic infection remains an unmet need.

Developing an HCV vaccine has proven challenging. Only two strategies have made it to human clinical trials in the past decade in HCV-negative individuals (2, 3). One trial assessed protection and showed that the vaccine failed at preventing chronic infection (2). The reason lies in the incredible diversity of HCV due to its error-prone RNA-dependent RNA polymerase, which is estimated to make about one error in every replicated genome (4). In addition to the 130,000 HCV sequences on the LANL database that include seven HCV genotypes, an eighth HCV genotype was recently characterized (5). Moreover, there are currently over 90 HCV subtypes. This vast diversity represents a formidable challenge for vaccine development because mutations in the viral envelope proteins (E1 and E2) have evolved to evade immunity. Additionally, the glycosylation of these proteins as well as association of the viral particles with circulating lipoproteins make the virus inaccessible to neutralizing antibodies. Passive immunization has been tested in animal models with some success, but the high genetic diversity of the virus limits the use of mAbs to only a small set of patients with antibody-sensitive viruses (6–8). Gaining insights into accessible E1E2 epitopes capable of inducing broadly neutralizing antibodies (bNAbs) that prevent reinfection is urgently needed for reinvigorating the development of HCV vaccines.

## bNAbs against HCV

Some people with HCV have a remarkable outcome: 15% to 45% of individuals who become infected spontaneously resolve the infection within a few months without establishing chronic, lifelong infection, and of those patients, 80% can clear subsequent reinfections (1). Several studies that examined the antibody response in acute and chronically infected patients demonstrated that bNAbs can be induced upon infection, with additional studies characterizing the binding epitopes of these bNAbs (9–11). However, how to best translate these findings into an efficient vaccine strategy remains unclear. A recent study by Underwood et al. in acutely infected patients showed that bNAb induction correlated with coinfection or recurrent infections with multiple distinct HCV subtypes, pointing to a multivalent vaccine strategy as a possible path forward (12). 

**Figure 1:**
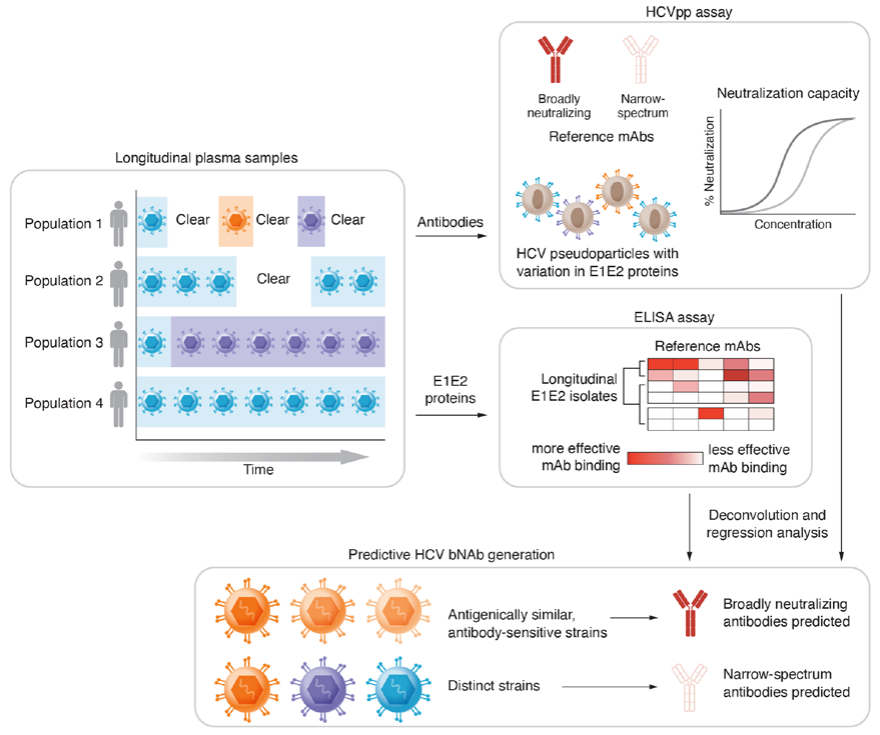
Characterization of the antibody response in HCV-infected patients. Frumento et al. (13) studied four different patient populations longitudinally. The HCV antibody response was evaluated using an HCVpp assay in which serological samples were compared with reference antibodies (mAbs) that had known neutralizing epitopes. They also performed an ELISA with reference mAbs to quantify antibody binding to E1 and E2 proteins derived from certain patient populations. Further regression analysis, deconvolution analysis, and hierarchical clustering revealed that neutralization breadth and potency correlated with viremia duration, multiple distinct infections, and specific antibody-sensitive epitopes. Antigenically similar HCV strains may associate with increased neutralizing antibody potency and breadth.

In this issue of the *JCI*, Frumento et al. describe how they took advantage of the information provided by studies of convalescent individuals (13). The authors focused on four key patient populations: (a) individuals who were infected and subsequently cleared multiple genetically distinct HCV strains (reinfection clearance); (b) individuals who cleared a primary infection, but did not clear a secondary infection with genetically distinct virus (reinfection persistence); (c) individuals who were sequentially infected with 2 distinct strains (persistence strain switch); and (d) individuals who were persistently infected with a single strain (persistence single strain). The authors characterized these patients serologically over 2 to 8 years to determine what conditions were critical in generating appropriate bNAb responses against HCV (Figure 1). It is important to note that this type of study is challenging, as many of the study population are unhoused and typically hard to follow consistently across such a long time scale (14, 15).

To examine neutralization capacity, the authors primarily utilized HCV pseudoparticles (HCVpp). This assay, developed in the early 2000s, is based on lentiviral particles pseudotyped with HCV E1 and E2 glycoproteins (16). The HCVpp system therefore allows the examination of HCV glycoprotein-dependent viral entry as well as the neutralization thereof with bNAbs. To characterize the breadth of antibody responses, the authors used an existing library of HCV E1 and E2 sequences from genotype 1, the most prevalent viral genotype in the US (17). Using sera from the different cohorts for neutralization, they found that the breadth and potency of neutralizing antibodies increased over the course of primary and secondary infections in more than 50% of the longitudinally collected samples. Surprisingly, the increase in antibody breadth and potency did not correlate with a positive chronic disease outcome, although it trended toward correlating with infection clearance. This result is potentially due to a small sample size, as previous studies have demonstrated such correlation (18, 19).

Next, the authors employed the HCVpp assay against 11 existing E1- and E2-specific mAbs with known neutralizing epitopes. By comparing the neutralization profiles between mAbs and plasma results, the authors successfully deconvoluted the type of antibodies present within the four patient groups (Figure 1). Data revealed that primary infections generated a variety of antibodies, both narrow spectrum and broadly neutralizing. Although narrow-spectrum antibodies were present during initial infections, bNAbs became dominant during subsequent infections. Importantly, the duration and number of distinct infections correlated with increased breadth and potency of neutralizing antibodies. However, examining the genetic distance (based on E1 and E2 sequences) between primary and secondary infections revealed that greater genetic distance was not associated with increased neutralization breadth and potency. This finding implies that repeated exposures to genetically divergent viruses are not required to generate more potent bNAbs, although the number of distinct infections with nondivergent genotypes correlated with increased antibody breadth and potency.

To address the conundrum that this result appeared different from that in earlier work, the authors added yet another data set to their analysis. They performed an ELISA to quantify the binding of longitudinally collected E1and E2 proteins from the four patient groups with reference mAbs. This strategy classified the antigenicity of the infecting viruses and revealed four antigenic E1E2 clades (Figure 1). Clades 1 and 3 were deemed antibody sensitive, while clades 2 and 4 were antibody resistant. While antigenic properties were similar between the viral E1 and E2 proteins from patients that eventually cleared the virus, the genotypes remained relatively distinct based on sequencing. Only genotypically distinct (while antigenically similar) infections from clade 1 were associated with increased neutralization. This finding suggests that a prime-boost vaccine strategy with repeated exposure to antigenically similar E1 and E2 proteins, rather than a multivalent approach with genetically diverse proteins from different subtypes, might better induce bNAbs. Interestingly, the authors observed that at the second or later infections, bNAb responses were directed mainly toward epitopes targeted by mAbs HEPC146, AR4A, HEPC74, and HEPC108. While not experimentally verified, this observation could be important in guiding vaccine development strategies toward these key neutralizing epitopes if confirmed.

## Conclusions and implications

The Frumento et al. (13) study was limited by the small sample size and lack of patient diversity. All patients were drug users and White; the majority were male and infected with genotype 1 HCV. T cell responses were not assessed in these subjects, but may have confounded the results given the small sample size.

Notably, most of the vaccine strategies developed for HCV to date focus on a single virus or a combination of viruses to maximize genetic diversity. The study by Frumento et al. (13) sheds light on why these vaccine strategies might have been unsuccessful. They propose, instead, a different approach in which repeated exposure to genetically distinct but antigenically similar HCV strains, based on observations of which repeated natural exposures, increased neutralizing antibody potency and breadth (Figure 1). Most importantly, the authors defined a set of epitopes recognized by four mAbs that are typically induced after repeated natural infections among those with more broadly neutralizing responses. These epitopes provide a key advance that researchers could directly apply in forthcoming vaccine trials. Although limited to genotype 1 viruses, the study creatively used clinical and in vitro generated data sets to computationally model the induction of bNAbs in HCV infection (Figure 1). Now, the stage is set to experimentally test the identified epitopes and the general concept of the proposed vaccine strategy. 

## Version 1 08/01/2022

Electronic publication
